# Upregulation of HLA Expression in Primary Uveal Melanoma by Infiltrating Leukocytes

**DOI:** 10.1371/journal.pone.0164292

**Published:** 2016-10-20

**Authors:** T. Huibertus van Essen, Sake I. van Pelt, Inge H. G. Bronkhorst, Mieke Versluis, Fariba Némati, Cécile Laurent, Gregorius P. M. Luyten, Thorbald van Hall, Peter J. van den Elsen, Pieter A. van der Velden, Didier Decaudin, Martine J. Jager

**Affiliations:** 1 Department of Ophthalmology, LUMC, Leiden, the Netherlands; 2 Department of Medical Statistics, LUMC, Leiden, the Netherlands; 3 Laboratory of Preclinical Investigation, Translational Research Department, Institut Curie, Paris, France; 4 Department of Clinical Oncology, LUMC, Leiden, the Netherlands; 5 Department of Immunohematology and Blood Transfusion, LUMC, Leiden, the Netherlands; 6 Department of Pathology, VU University Medical Center, Amsterdam, the Netherlands; 7 Department of Clinical Hematology, Institut Curie, Paris France; Universitat Hohenheim, GERMANY

## Abstract

**Introduction:**

Uveal melanoma (UM) with an inflammatory phenotype, characterized by infiltrating leukocytes and increased human leukocyte antigen (HLA) expression, carry an increased risk of death due to metastases. These tumors should be ideal for T-cell based therapies, yet it is not clear why prognostically-infaust tumors have a high HLA expression. We set out to determine whether the level of HLA molecules in UM is associated with other genetic factors, HLA transcriptional regulators, or microenvironmental factors.

**Methods:**

28 enucleated UM were used to study HLA class I and II expression, and several regulators of HLA by immunohistochemistry, PCR microarray, qPCR and chromosome SNP-array. Fresh tumor samples of eight primary UM and four metastases were compared to their corresponding xenograft in SCID mice, using a PCR microarray and SNP array.

**Results:**

Increased expression levels of HLA class I and II showed no dosage effect of chromosome 6p, but, as expected, were associated with monosomy of chromosome 3. Increased HLA class I and II protein levels were positively associated with their gene expression and with raised levels of the peptide-loading gene *TAP1*, and HLA transcriptional regulators *IRF1*, *IRF8*, *CIITA*, and *NLRC5*, revealing a higher transcriptional activity in prognostically-bad tumors. Implantation of fresh human tumor samples into SCID mice led to a loss of infiltrating leukocytes, and to a decreased expression of HLA class I and II genes, and their regulators.

**Conclusion:**

Our data provides evidence for a proper functioning HLA regulatory system in UM, offering a target for T-cell based therapies.

## Introduction

Uveal melanoma (UM) is the most common primary intraocular tumor in adults with an incidence of 5.1 per million [1]. Metastases develop in up to 50% of patients [2], and there is still no effective cure for metastatic disease. Once the tumor has metastasized, the median patient survival is approximately one year, and survival has remained largely unaltered during the last 40 years [1]. A patient’s individual prognosis is easily predictable, as during the last three decades several reliable prognostic markers have been found for UM and tests predicting patient survival have been developed. These include gene-expression profiling (GEP) [3], and chromosome testing: patients that have lost one copy of chromosome 3 (monosomy 3) are at high risk to die from metastatic disease [4,5]. Other chromosomal aberrations which modulate the risk of metastasis are 6p gain [6,7] and 8q gain [8–10]. Gain of 6p is only rarely observed in the same tumor as monosomy 3, and is associated with a favorable prognosis, while gain of 8q is often associated with monosomy of chromosome 3 and associated with a bad prognosis [6,7].

Inflammation is another prognostic marker for the development of metastatic disease [4,11], and is associated with monosomy 3 [4,12]. Inflammation has been established as one of the hallmarks of cancer [13], and despite the fact that the eye is an immune-privileged site, inflammation can be present within the intraocular tumor. The inflammatory phenotype seen in UM is characterized by an increased number of tumor-infiltrating lymphocytes and macrophages, as well as an increased expression on UM cells of Human Leukocyte Antigens (HLA) class I and II [4]. Several studies already showed that, in contrast with many other malignancies, a high expression of the HLA class I and II antigens is associated with a poor prognosis [14–16]. The sensitivity of UM to natural killer (NK)-cell mediated lysis was shown to be inversely correlated to this HLA protein expression [15,17]: UM cells with a high HLA class I expression gave rise to higher numbers of metastases in mice, which suggests that the tumor cells are able to escape from NK-cell mediated lysis. As especially UM with a high HLA expression give rise to metastases in patients, this possible escape mechanism may also be present in humans [18].

The genes encoding both the HLA class I and II antigens are located on chromosome 6p. This faces us with an intriguing paradox because in general, chromosomal gain tends to lead to an increased gene expression in tumors [19,20]. While in UM, 6p gain is associated with a good prognosis, an increase in HLA expression of both classes is associated with a poor prognosis. This made us wonder what exactly regulates HLA expression levels in UM: chromosomal dose effects (gain or loss of chromosome 6), an intrinsic genetic regulation, or external influences. We know that occasionally specific HLA alleles are not expressed [21]. Therefore we looked in a previous study at the association between HLA expression and LOH (Loss of Heterozygosity) of chromosome 6 [22]. However, no relation between LOH and lack of expression was observed at the time, suggesting that a low HLA expression was not caused by loss of chromosome 6 material. Furthermore, several studies have shown that the more malignant UM are not characterized by a downregulation, but by an upregulation of HLA class I and II antigens [14–16].

While expression at the cell surface depends on a properly-functioning peptide-loading system, regulation at the gene level depends on a set of different genes, which includes *NLRC5* and *CIITA*. *NLRC5* plays a crucial role in the transcriptional regulation of HLA class I genes [23], and *CIITA* in the transcriptional regulation of the *HLA class II genes* [24], while it is also involved in *HLA class I* transcriptional activation [25]. The promoters *NLRC5* and *CIITA* are on their turn under the influence of, amongst others, the interferon-regulatory factor 1 (IRF1) [26]. Furthermore, Holling et al., using UM cell lines, reported that HLA class II could be induced in half of theirUM cell lines, and showed that the lack of HLA class II expression in one particular cell line was caused by epigenetic silencing of the gene encoding CIITA. Silencing of *CIITA* was mediated through EZH2 (Enhancer of Zeste Homologue 2, a Polycomb Repressive Complex 2 subunit; chr7q), which triple methylates lysine 27 in histone H3 [27]. That not only transcriptional regulators influence HLA class I and II expression, but also external influences, was also shown using UM cell lines where interferon-gamma (IFNG) stimulation led to increased levels of HLA class I and II [27,28]. Additionally, downregulation could be induced by tumor growth factor beta (TGFB) [17,28]. However, all of these studies regarding the regulation of HLA expression were performed on a limited number of available cell lines. As IFNG induces upregulation of HLA molecules in cell lines *in vitro*, IFNG produced by tumor-infiltrating leukocytes may have a similar effect *in vivo* in high-risk UM. HLA expression is of great importance for T-cell based therapies, because without HLA-molecules, T-cells cannot react to and subsequently destroy their target cells [29]. Therefore it is important to determine whether HLA expression in UM cells functions properly, and how it is regulated.

We here investigate whether chromosomal dose effects or specific known regulators influence HLA gene or protein expression in UM, this time studying primary enucleated tumors and not cell lines. We analyze the relationship between HLA class I and II RNA and protein levels, and genes involved in the regulation of (HLA) transcription, genes of the peptide-loading system, such as the TAP molecules, and the influence of the absence or presence of one chromosome 3 or 6p. Additionally, we assess the association and influence of the microenvironment on HLA gene expression by comparing expression levels in human primary or metastatic UM with their corresponding xenografts placed in mice, which lack tumor-infiltrating leukocytes.

## Methods

### Study Population

Tumor tissue was obtained from 28 eyes that underwent primary enucleation for UM between 1999 and 2004 at the LUMC in Leiden, the Netherlands. Patient records and survival were updated from the patient’s charts and through the Dutch National Registry; the last update was in November 2013.

The current study population has previously been described [30], but only cases with material suitable for gene-analysis were included; each tumor sample was processed for conventional histopathological evaluation, including cell type assessment according to the modified Callender classification. The mean age at enucleation of the 28 patients was 62 years (median 68, range 28–84 years) and median follow up was 72 months (median 76, range 14–145 months). At the time of the study, 13 patients were alive, 13 had died due to UM metastases, and in two cases, the cause of death was unknown. The mean time to metastasis was 37 months (median 30, range 14–96 months).

### SNP and gene expression

A genome wide micro-array analysis on single-nucleotide polymorphisms (SNPs) was performed with the Affymetrix 250K Nsp array (Affymetrix, Santa Clara, CA, US) to acquire a highly detailed image of the chromosome copy numbers. The average number of copies of all genes lying on the short (p) or long arm (q) of chromosome was used to determine gain or loss of that arm. For chromosome 6p we focused on gain or loss of the HLA region on that arm.

Gene-expression profiling was performed with the Illumina HT12 v4 array (Illumina, Inc., San Diego, CA, US) for the HLA genes (*HLA-A*, *-B*, *beta-2-microglobulin*, *HLA-DR [HLA-DRA*, *HLA-DRB1*, *HLA-DRB3*, *HLA-DRB6]* and *HLA-DQ [HLA-DQA1*, *HLA-DQB1]*), the genes encoding HLA transcriptional regulators (*NLRC5* [25,26,31], *CIITA* [25,26,31], *IRF8*, *IRF1*, *IRF2*and genes of the peptide-loading machinery (*TAP1*, *TAP2*, *PDIA3*, *Tapasin*, *Calreticulin*). Beta-2-microglobulin (B2M) is a component of the HLA class I complex at the cell surface, and should theoretically be upregulated when there are more HLA-molecules on the cell surface. However, the B2M gene is located on a different chromosome (15q21). HLA-C was not analyzed, as it has a tenfold-lower expression at the protein level than HLA-A and -B [32,33]. As the peptide-loading machinery is needed to get a functional HLA class I molecule expressed at the cell surface, we looked at several of these genes. In cases where Illumina provided multiple probes for the same gene, the mean value of these probes was used for further analysis. One of four probes for HLA-A (ILMN_2165753) was omitted because of discordancy with the values of the other three HLA-A probes.

The Illumina array data discussed in this publication have been deposited in NCBI's Gene Expression Omnibus [34] and are accessible through GEO Series accession number GSE84976 (https://www.ncbi.nlm.nih.gov/geo/query/acc.cgi?acc=GSE84976)."

### Validation

Quantitative polymerase chain reaction (qPCR) was performed, as described previously [35], in duplicate on the selected genes for HLA and HLA-regulation activators, as a validation of the results found with gene-expression profiling.

In short, RNA was extracted from the fresh frozen primary tumors using an RNeasy Mini Kit (Qiagen). Complementary DNA (cDNA) was synthesized with the iScript cDNA synthesis kit (Bio-Rad). Beacon Designer (Biosoft) was used for primer design. Primers for *ACTB (β-actin;* OMIM 102630), *GAPDH* (OMIM 138400), *RPL13* (OMIM 113703), and *RSP11* (OMIM 180471) were included for selecting suitable reference genes. Gene expression was calculated by normalizing the C-value of each marker to the reference genes. Reference genes were selected based on their stable expression in the tissue, which was measured with the geNorm Software [36].

In the material studied, *RPL13* and *RPS11* were stably expressed, and the genes of interest were corrected for the geometric mean of these two reference genes as described previously [37].

For a overview of the primers used, see the supplements (S1 Table).

### Immunohistochemistry

Immunohistochemical staining for HLA class I (HLA-A, -B) and II (HLA-DR) was previously performed as described [4]. In short, immunohistochemical staining was carried out using the mouse monoclonal antibodies HCA2 (Produced by the Netherlands Cancer Institute, Amsterdam, the Netherlands) exclusively staining HLA-A heavy chains, HC10 (Produced by The Netherlands Cancer Institute, Amsterdam, the Netherlands) which binds to HLA-B and -C heavy chains [38,39], and Tal.1B5 (DakoCytomation, Glostrup, Denmark) reacting with HLA-DR alpha chains. The number of tumor cells positive for each marker was counted at 100X magnification and expressed as percentage of the total number of tumor cells (S2 Table).

Immunofluorescence staining for infiltrating macrophages was performed and described previously, with antibodies against CD68 (clone 514H12; Abcam, Cambridge, UK) and CD163 (clone 10D6; Novocastra, Newcastle-upon-Tyne, UK), and expressed as pixels per millimeter squared [40]. T cells were identified and scored previously using an anti-CD3 antibody (ab828; Abcam), and expressed as numbers of cells per millimeter squared [12].

### Xenografts

Fresh intraocular or metastatic human tumor samples were acquired with informed consent at the Institut Curie (Paris, France), and immediately transplanted into the interscapular fat pad of two to four non-preirradiated immunodeficient female SCID mice, and considered to be stable xenografts for characterization after three consecutive mouse-to-mouse passages [41]. Total RNA was isolated from frozen material of the fresh intraocular and metastatic human tumor samples, and from frozen material of the corresponding xenografts, and subsequently analyzed with GeneChip Human Exon 1.0 ST microarrays (Affymetrix) [42].

Of these tumors, eight of primary and four of metastatic origin were included in our study and expression on the microarray was analyzed for *HLA-A*, *HLA-B*, *HLA-DR (HLA-DRA)*, and *HLA-DQ (HLA-DQA1*, *HLA-DQA2)*, and markers of infiltrating T cells: *CD3 (CD3D*, *CD3E)*, *CD4)*, and *CD8 (CD8A*, *CD8B)*, and macrophages: *CD68*, and *CD163*.

### Statistics

Data analysis of the material obtained at the LUMC in Leiden was performed with the statistical programming language R version 3.0.1 (R: A Language and Environment for Statistical Computing, R Core Team, R foundation for Statistical Computing, Vienna, Austria, 2014, http://www.R-project.org), supplemented with specialized packages for SNP and RNA analysis. The main package used for SNP analysis was aroma.affymetrix [43–45], supported by ‘DNAcopy’ (Venkatraman E. Seshan and Adam Olshen, DNAcopy: DNA copy number data analysis. R package version 1.34.0), ‘sfit’ (Henrik Bengtsson and Pratyaksha Wirapati (2013), sfit: Multidimensional simplex fitting. R package version 0.3.0/r185, http://R-Forge.R-project.org/projects/matrixstats/), and ‘R.utils’ (Henrik Bengtsson (2014), R.utils: Various programming utilities, R package version 1.29.8, http://CRAN.R-project.org/package=R.utils). Data of eighty-four healthy controls served as reference set, obtained with the same Affymetrix 250K Nsp chip (Affymetrix, Santa Clara, CA, USA) by the Department of Human Genetics at our center. The ‘Aroma.Affymetrix’ package made it possible to use these SNP microarrays to determine copy number values [43–45] The packages used for RNA microarray analysis were ‘limma’ version 3.16.8 and the specific packages for Illumina microarrays: ‘lumi’ version 2.12.0, ‘annotate’ (R. Gentleman, annotate: Annotation for microarrays, R package version 1.38.0), and ‘illuminaHumanv4.db’ (Mark Dunning, Andy Lynch and Matthew Eldridge, illuminaHumanv4.db: Illumina HumanHT12v4 annotation data (chip illuminaHumanv4), R package version 1.18.0).

Data of the xenografts and corresponding original human tumors were analyzed at the Institut Curie (Paris, France) with R software (version 2.12) at gene level using custom Brain array Chip Description Files (CDF) based on Entrez Gene database (version 13) as described in detail by Laurent et al. [42]. For this study, a paired sub-analysis was made at the Institut Curie with regard to the genes of our interest.

All other statistical analysis was performed with SPSS 20.0.1 (IBM SPSS Statistics, IBM Corporation, Armonk, NY, US). Cumulative survival was calculated with Kaplan-Meier and its significance analyzed with the log rank test. Hazards ratios (HR) were measured with Univariate Cox regression models. Associations for clinical categorical variables with gene expression were determined using the Mann-Whitney U test (Wilcoxon rank sum test) for non-parametric analysis. Statistical correlation between two continuous variables was calculated with the Spearman’s rank test. For survival analysis with Kaplan-Meier, the linear variables were dichotomized at 50% cut off, unless mentioned otherwise. Statistical significance was assumed for a p-value <0.05.

### Study approval

The collection of material and the research performed with it for this study has been agreed upon by the Medical Ethics Committee of the LUMC (Leiden University Medical Center, Leiden, the Netherlands). The research protocol adhered to Dutch law and the current version of the tenets of the Declaration of Helsinki (World Medical Association of Declaration 1964; ethical principles for medical research involving human subjects). Patients were informed about the use of their enucleated eyes for research, and signed an informed consent prior to the enucleation. The performance of the xenograft study was in accordance with the French Ehtics Committee (Ministère de l'Alimentation, de l'Agriculture et de la Pêche, Direction de la Santé et de la Protection Animale, Paris, France). Both studies were thus specifically approved by the Ethics Committee of the respective centers, the first one being the Medical Ethics Committee of the LUMC (Leiden University Medical Center, Leiden, the Netherlands) and the second one being the just mentioned French Ethics Committee (Ministère de l'Alimentation, de l'Agriculture et de la Pêche, Direction de la Santé et de la Protection Animale, Paris, France).

## Results

### HLA gene expression and HLA immunohistochemistry

We wondered whether differences that were noticed in *HLA* gene-expression assays correlated with previous immunohistochemical staining results on tumor cells and observed that the gene-expression of *HLA-A* and *-B* was significantly correlated with the percentage of tumor cells being, respectively, positive for HCA2 (HLA-A) and HC10 (HLA-B/C) (Table 1). *B2M* gene-expression correlated positively with HCA2 and HC10 staining too, although not significant for HCA2. Gene-expression of *HLA-DR* failed to correlate with monocolonal antibody Tal.1B5. Gene-expression levels of the HLA molecules in the Illumina test correlated with death due to metastases (S3 Table). When comparing clinical and histopathological parameters, gene expression of *HLA-A*, *HLA-B*, *B2M*, and *HLA-DR* was positively associated with age at enucleation and with the largest basal diameter of the tumor, a well-known adverse parameter.

**Table 1:** Correlation of gene-expression with immunohistochemistry for HLA.

	mAb HCA2		mAb HC10		mAb Tal.1B5	
Gene-expression (Illumina)	(HLA-A)		(HLA-B/C)		(HLA-DR)	
	*r*	*p*	*r*	*p*	*r*	*p*
*HLA-A*	.434	0.02	.545	0.003	.161	0.41
*HLA-B*	.488	0.01	.609	0.001	.286	0.14
*B2M*	.346	0.07	.508	0.006	.279	0.15
*HLA-DR*	.242	0.21	.332	0.08	.225	0.25
*HLA-DQ*	.301	0.12	.400	0.04	.172	0.38

Correlation of HLA gene-expression, as determined with the Illumina array, with the results of HLA immunohistochemical staining in 28 cases of UM. r = two-tailed Spearman correlation coefficient. p = p-value.

The genes that code for the HLA class I and II antigens are located on chromosome 6p, which has prognostic importance in UM. In order to determine whether the level of HLA class I and II antigens was influenced by a gene-dosage effect, we determined the presence of chromosome 6 by SNP array, and compared HLA and B2M gene expression levels with chromosome dosage. Additionally, we examined the gene-dose effect of chromosome 3, as a previous study from our laboratory had indicated an association between loss of chromosome 3 and increased HLA expression levels on primary UM(8). An overview of the clinical, chromosomal, gene expression, and histological data is provided in S4 Table.

### Aberrations of chromosome 3 and 6p

Aberrations of chromosome 3 and 6p were analyzed with a SNP micro-array in 28 UMs. Monosomy 3 was present in fourteen (50%) cases and associated with death due to metastases (Kaplan-Meier, *p* < 0.001). Gain of the HLA region of chromosome 6p was present in eight cases (29%), and associated with good survival (Kaplan-Meier: *p* = 0.049). Gain of 6p co-occurred with monosomy 3 in only one case.

To determine a possible dose effect of chromosome 6 on HLA expression levels in UM, we investigated whether there was an association of chromosome 6p dose and gene or protein expression of HLA. Monosomy of chromosome 3 was associated with an increased gene expression of *HLA class I* and *B2M* but not of *HLA class II*. Gain of 6p occurred almost exclusively in tumors without monosomy 3, and when looking at all tumors, an association was observed between 6p gain and less *HLA-B* expression (*p* = 0.049). Expression of HLA class I and II as determined by immunohistochemistry was not associated with 6p gain (HLA-A *p* = 0.12; HLA-B/C *p* = 0.26; HLA-DR *p* = 0.24).

**Fig 1:**
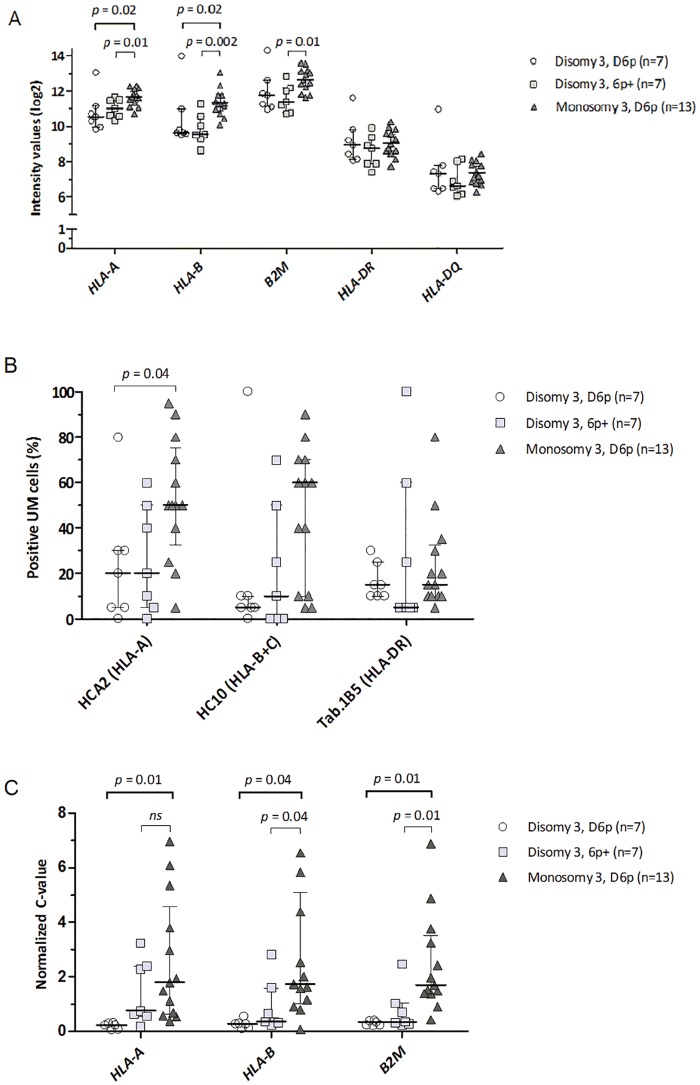
Chromosomal aberrations and HLA expression. Comparison between chromosomal aberrations and the expression of HLA class I and II antigens in a set of 27 primary UM. Tumors are divided according to their chromosome 3 and 6 status (disomy or monosomy of chromosome 3, and disomy of chromosome 6 or gain of 6p). HLA gene expression was determined using an Illumina microarray (A) and protein expression by immunohistochemistry (B) in UM. Additionally, HLA gene expression was determined using qPCR, which served to validate the Illumina findings (C). Four data points of the qPCR that are outside the axis limits (> 11 and < 24) are not shown (HLA-A, D3D6p: 17; HLA-B, D3D6p: 24, and M3D6p: 12; B2M, D3D6p: 13). Only significant p-values are shown, all other comparisons between the groups were not significant (p-values not shown). Error-bars represent the interquartile range. Results were obtained using the Mann-Whitney U tests.

However, the effect of the loss of one copy of chromosome 3 may have overshadowed that of chromosome 6p. Therefore we separated monosomy 3 and disomy 3 tumors into those with or without gain of 6p. As the group with monosomy 3 with gain of 6p consisted of only one tumor, we did not use this case for analysis. As expected, monosomy 3 tumors showed a higher expression of HLA class I than disomy 3 tumors, at the gene-expression as well as the protein-expression level (Fig 1A and 1B). When looking at the effect of 6p gain in disomy 3 tumors, no significant differences in gene and protein expression were observed between tumors with a normal 6p (n = 7) or a gain of 6p (n = 7). Similarly, HLA class II gene and protein expression was not different as well. We can conclude that there is no clear dose effect of chromosome 6p on HLA class I and II expression.

### Peptide-loading genes and HLA transcriptional regulators

As chromosomal gain of 6p had no influence on the expression levels of *HLA* genes, we questioned whether HLA expression was influenced by the peptide-loading machinery or any of the known HLA regulators.

We compared expression levels of the *HLA class I* subtypes with expression of the genes encoding the main molecules of the peptide-loading machinery, eg. with *TAP1*, *TAP2*, *tapasin* and *calreticulin*. *TAP1*-gene expression correlated positively with the gene-expression levels of *HLA class-I* and *B2M*, while *TAP2* in general showed a similar correlation, although this was not significant for all comparisons (Table 2). The other antigen-presenting machinery molecules were not co-regulated with HLA antigen expression.

**Table 2:** Correlation between *HLA-* gene expression and the expression of the genes encoding the HLA transcriptional regulators and peptide-loading machinery.

	*HLA-A*		*HLA-B*		*B2M*					
Peptide loading machinery	*r*	*p*	*r*	*p*	*r*	*p*				
*TAP1*	.859	<0.001	.809	<0.001	.814	<0.001				
*TAP2*	.367	0.06	.350	0.07	.465	0.01				
*PDIA3*	.204	0.30	.184	0.35	.159	0.42				
*Tapasin*	.171	0.38	.135	0.50	-.038	0.85				
*Calreticulin*	-.036	0.86	.031	0.88	-.218	0.27				
	*HLA-A*		*HLA-B*		*B2M*		*HLA-DR*		*HLA-DQ*	
HLA transcriptional regulators	*r*	*p*	*r*	*p*	*r*	*p*	*r*	*p*	*r*	*p*
*CIITA*	.276	0.16	.407	0.03	.408	0.03	.415	0.03	.465	0.01
*NLRC5*	.280	0.15	.470	0.01	.389	0.04	.557	0.002	.492	0.01
*IRF1*	.758	<0.001	.854	<0.001	.853	<0.001	.828	<0.001	.852	<0.001
*IRF2*	.137	0.49	.096	0.63	.179	0.36	.032	0.87	-.085	0.67
*IRF8*	.578	0.001	.679	<0.001	.689	<0.001	.937	<0.001	.916	<0.001

*Correlations based on Illumina array data*. *r* = *two-tailed Spearman correlation coefficient*. *p* = *p-value*.

We subsequently focused on the influence of known regulators of HLA gene expression. NLRC5 is a regulator for *HLA class I* and not for *HLA class II* genes, while CIITA is essential for the transcriptional regulation of HLA class II genes. In contrast to NLRC5, CIITA also plays an ancillary function in the transcriptional regulation of HLA class I genes. Other known HLA regulators, influencing both class I and II, are IRF1, IRF2, and IRF8. When we compared expression of these HLA transcriptional regulators with HLA expression in tissue of human uveal melanoma, we observed that the gene-expression levels of the HLA transcriptional activators *IRF1* and *IRF8* correlated positively with *HLA class I* and *B2M*, *NLRC5* with *HLA-B* and *B2M* expression, and *CIITA* with *HLA-B* as well as with *B2M* (Table 2). Additionally, the gene-expression levels of *IRF1*, *IRF8*, *NLRC5* and *CIITA* correlated positively with the expression levels of both *HLA class II* subtypes, i.e. *HLA-DR* and *HLA-DQ*. IRF1 and IRF8 exert their function on HLA class II genes via their involvement in CIITA transcription.

Only an increased gene-expression level of *IRF1* was associated with monosomy 3, expression levels of *IRF2*, *IRF8*, *NLRC5*, and *CIITA* were not associated (S1 Fig).

A high expression of *TAP1* and of *IRF1* was associated with death due to metastases (*TAP1*: Univariate Cox regression: HR = 1.6, *p* = 0.004, and Kaplan-Meier: *p* < 0.001; *IRF1*: Univariate Cox regression: HR = 1.4, *p* = 0.06, and Kaplan-Meier: *p* = 0.007). *NLRC5*, *CIITA*, *IRF2*, and *IRF8* displayed no association with survival.

### Validation

Quantitative polymerase chain reaction (qPCR) on the same 28 UMs was performed to validate our findings based on the gene-expression values obtained with Illumina HT12 v4 array for HLA class 1 genes (Fig 1C). Unfortunately, a suitable primer pair for qPCR could not be developed for *IRF8*. qPCR confirmed most of the correlations between the *HLA* genes and *TAP1*, *CIITA*, *IRF1*, and *NLRC5* (Spearman correlations, S5 Table). Using qPCR data, *TAP2* was significantly correlated with the *HLA* genes.

### Tumor-infiltrating immune cells

**Fig 2:**
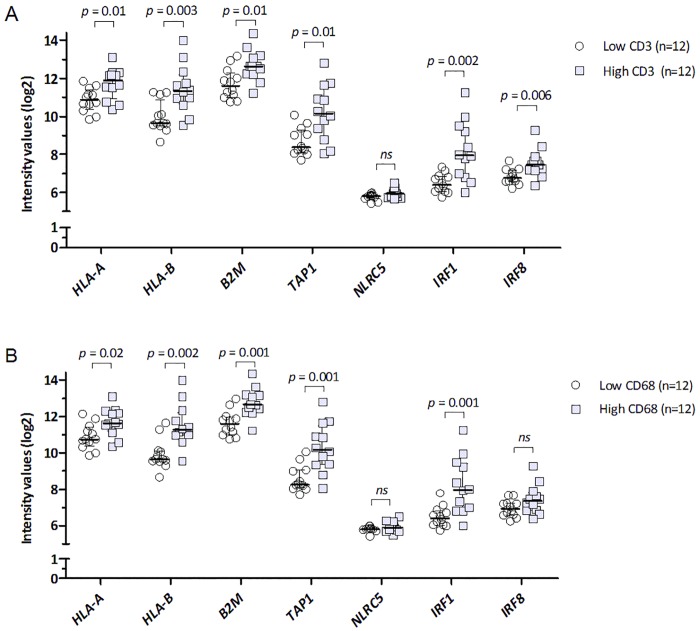
Tumor infiltrating immune cells. Association between a low or high density of tumor-infiltrating CD3+ (A), and CD68+ (B)cells and several HLA and HLA-related genes (Illumina array) in primary UM. CD3 (cells/mm^2^) and CD68 (pixels x 10^3^/mm^2^) scores were dichotomized at the median.

Expression of several HLA class 1 and 2 molecules, regulators of the antigen-presenting machinery, as well as several of the transcriptional regulators, are thus co-regulated in human UM samples, and the presence of most of these, showed an association with loss of one chromosome 3. We noticed that all of these molecules are part of the interferon-regulated pathway. As infiltrating cells are considered important sources of interferon and infiltrating leukocytes are also an important part of the inflammatory phenotype of UM, we compared HLA expression with infiltrating leukocyte density, using the Illumina *HLA* expression data. We looked at the presence of macrophages and CD3+ T-cells, as markers of the presence of an immune infiltrate. We separated the tumors into two groups, with a low or high infiltration, and compared expression levels of *HLA-A*, *HLA-B*, *B2M*, *TAP1*, *IRF1*, *IRF8* and *NLRC5* between these two groups. Overall, a high infiltrate was associated with increased expression of the genes related to HLA class I (Fig 2A and 2B).

### Xenografts

**Fig 3:**
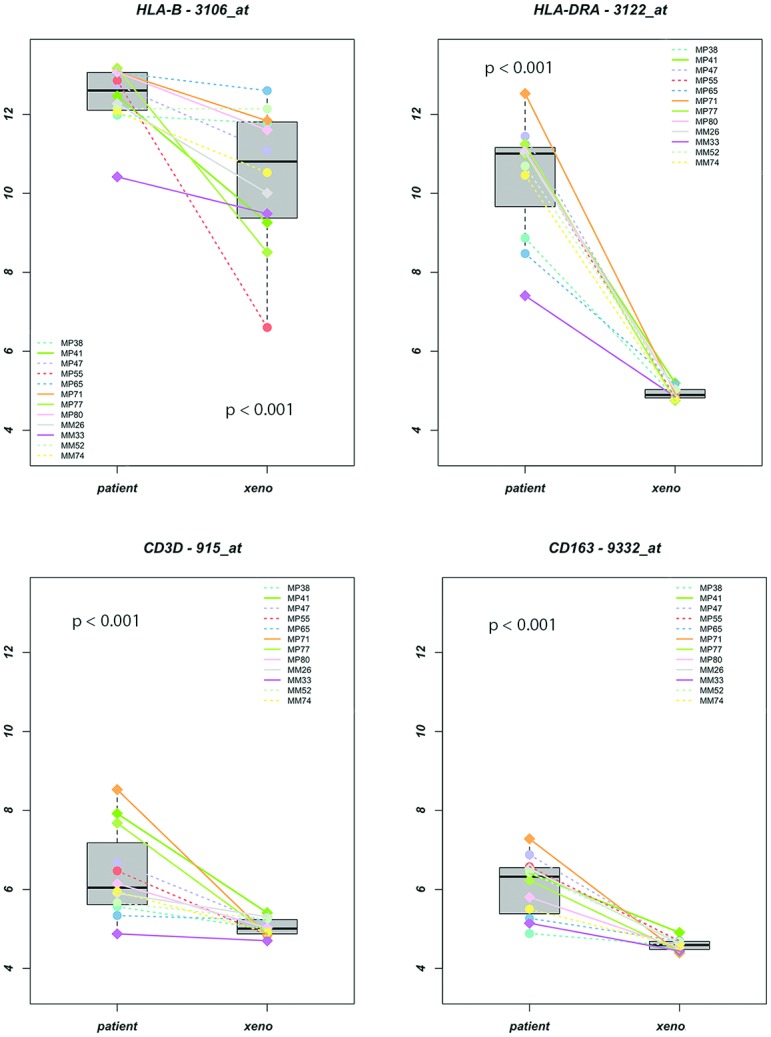
Effect of the absence of human leukocytes. Gene-expression (log 2 intensity values) of HLA-B, HLA-DRA, CD3D (as marker for T-cells), and CD163 (as marker for macrophages), of the original tumors (patient) compared to the xenografts (xeno). MP’s are primary tumors; MM’s are metasisis.

The clear association between the presence of infiltrating leukocytes and *HLA-A*, *HLA-B*, *TAP1*, as well as the HLA regulator genes suggests that the presence of leukocytes is associated with the level of expression of all HLA-associated molecules. However, it may be that a high HLA expression attracts leukocytes to the site of inflammation, or it may be that the HLA expression is upregulated by cytokine production from the leukocytes. These two options can be tested by comparing intraocular UM with xenografts from the same tumors in mice. When human tumors are placed as xenografts into SCID mice, they loose their infiltrating human leukocytes over time due to their limited timespan [46], and any environmental effect caused by these leukocytes disappears. If the HLA expression is upregulated due to leukocyte-produced interferon, its expression should go down in a xenograft. Twelve xenografts, consisting of eight primary tumors and four metastases of UM, were analyzed and their gene expression levels were compared with the original human tumor tissue. This allowed us to compare the effect of the absence of infiltrating human leukocytes on the expression of the genes of our interest (Fig 3). Indeed, a decrease in infiltrating T cells was observed (*Cd3E p* < 0.001, *Cd3D p* <0.001, *Cd4 p* < 0.001, *Cd8A p* = 0.001, Cd8b *p* = 0.34). Also, the markers for macrophages, *Cd68* (*p* < 0.001) and *Cd163* (*p* < 0.001), were decreased in xenografts. In parallel, the xenografts showed a decrease of the HLA genes investigated, as well as of most of the peptide-loading machinery genes and transcription regulation genes (S6 Table).

## Discussion

We found that the immune infiltrate was associated with an increased HLA-gene expression in UM. Immune infiltrate is associated with monosomy 3 [4,12], and loss of infiltrating human leukocytes from the xenografts in SCID mice resulted in a lower HLA class I and II gene expression. Many of the studies on UM focus on markers predicting prognosis [3,5,7,18,40]. Predicting prognosis, important as it is, does not explain why and how these markers are related to an altered life expectancy. We first compared the relationship between the expression of HLA genes and aberrations of chromosome 3 and 6p, containing the HLA genes, on 28 extensively documented UM tumors. We observed that for the *HLA class I* and *II* genes, an increased number of copies of chromosome 6p led not to a higher HLA expression, showing that UM deviate from the general rule that gain of gene copy number leads to a higher expression of those genes (Fig 1).

Following the line of HLA production from the presence of its complexes on the cell membrane downwards to the amount of RNA (gene-expression) and finally to the genes located in the nuclei, two patterns could be discerned: HLA class I gene and protein levels were not related to the dose effect of chromosome 6p, yet they were clearly and inversely associated with the dose of chromosome 3. This not only affected the HLA class I and II antigens, but also transporter molecules, such as B2M located on a different chromosome than 6, as well as HLA expression regulators. In our earlier work on the same tumors [4,12,22], we had used karyograms and FISH analysis on isolated nuclei for determining the presence of monosomy 3, while in the current study we used the more precise SNP array. We here show that loss of one chromosome 3 is associated with an increased leukocytic infiltrate, and increased expression of not only the HLA class I and II molecules, but also of molecules related to the peptide-loading machinery and transcriptional regulators.

The apparently intact HLA protein production tree in UM directed us to look outside the cell for factors influencing HLA expression. The HLA transcriptional regulators are under the influence of IFNG, a cytokine often produced by lymphocytes [47]. IFNG can activate Janus kinase 1 and 2 (JAK1 and JAK2), which causes activation of STAT1 (signal transducer and activator of transcription 1) and IRF1, leading to induction of CIITA [26]. With regard to HLA class I expression, NLRC5 has been identified as the key transcriptional regulator at this level in human cells of different origin (embryonic kidney cells, HeLa cells, and Jurkat T cells) [23]. Our current findings have been obtained on a set of 28 primary UM, and show that not only TAP1 is being upregulated together with HLA, but that *HLA class I* is upregulated together with *TAP1*, *IRF1*, *IRF8*, *NLRC5* and *CIITA*, and *HLA class II* together with *CIITA* and *IRF1*, which agrees with the previous results obtained using cell lines [27].

It was previously reported that EZH2 (also known as KMTase Enhancer of Zeste Homolog 2), a member of the polycomb repressive complex 2 (PRC2), is involved in blocking IFNG-induced upregulation of *CIITA* in a UM cell line [27]. Monosomy 3 in uveal melanoma is associated with loss of BAP1 (BRCA1 associated protein-1; chr3p), [8,48], which is connected to PRC2 (of which EZH2 is a member) through ASXL1(additional sex combs-like transcriptional regulator 1) [49,50]. This could represent a possible connection between BAP1 and expression of HLA in uveal melanoma, as study in mice showed that loss of BAP1 leads to increased levels of EZH2 (and PRC2) with repressed expression of its targets [51], including thus CIITA, leading ultimately to less HLA class II expression. However, this is not what we found in uveal melanoma, where BAP1 loss seemed to be associated with increased HLA expression [52].

The general picture that emerges from the Illumina array was validated with the qPCR (Fig 2). *HLA class I* and *HLA II* are upregulated in monosomy 3 tumors, and the upregulation of HLA class I genes is positively correlated to *IRF1*, *CIITA*, and *TAP1* (and *TAP2*), while *HLA class II* is upregulated together with *IRF1*, *CIITA* and *NLRC5*. *NLRC5* is located on the same chromosome as *CIITA* (chromosome 16). Since both of these transcriptional regulators belong to the NOD-like receptor family of proteins and share some regulatory elements in their promoters, it could therefore be a co-regulatory effect that explains this finding for NLRC5. Some UM are known to loose copies of Chromosome 16 [53], so likely here is no gene dose effect.

As we were not finding clues indicating that the regulation of HLA expression was internally deranged in these UM, we aimed to find the origin of the elevated HLA expression by factors outside the UM cells. The most well-known factor is IFNG, which has previously been shown to stimulate HLA expression in UM cell lines [28], IFNG is predominantly secreted by CD4+ T helper 1 cells (Th1), CD8+ cytotoxic T cells, and NK cells, although it is also to a lower degree produced by professional antigen-presenting cells (APCs), including macrophages, and B-cells. Our data confirmed that indeed a high leukocyte infiltration of CD68+ and CD3+ leukocytes, was associated with an increase in gene expression of *HLA*, *B2M*, *TAP1*, and *IRF1*. Some of these markers may be more highly expressed at the RNA level due to the presence of leukocytes in the RNA that was used for the array and qPCR. One may suggest that the PCR data identified HLA antigens on infiltrating cells, however all original studies were performed using immunohistochemistry on tumor sections, analyzing melanoma-cell specific HLA class I and II and B2M expression. We can thus conclude that we are really looking at the HLA expression of the tumor cells themselves.

Using SCID-mice (lacking T and B cells) into which freshly-obtained UM were grafted, we observed that xenografts obtained from tumors that originally showed a high *HLA class I and II* expression, showed a much lower *HLA class I and II* expression than their original counterpart (Fig 3). The original tumors (primary and metastatic) were usually monosomic for chromosome 3 and exhibited high gene-expression levels of *CD3*, *CD4*, *CD8* (T-cell markers), and *CD68* and *CD163* (macrophages markers), as well as a high HLA class I and II gene expression. The average expression of all of these genes decreased in the xenografts, although the tumors continued to show monosomy of chromosome 3. This means that the tumor-infiltrating lymphocytes (TILs), and especially the T cells as main producers of IFNG, are the ones triggering the increased HLA expression in UM. Jehs et al. observed that UM cell lines are not producing IFNG themselves, and that upon co-culture with IFNG-secreting T cells, the UM cells were responding by synthesizing chemokines that create a tumor-promoting environment with increased HLA expression and attraction of M2 type macrophages [54]. Indeed, the T cell-containing primary UMs as well as the xenografts contained high numbers of macrophages. Following xenografting, a decrease in gene-expression of *Cd68* was observed.

Although the question remains how these T cells are exactly recruited to the tumor, and whether they do or do not recognize the peptides presented in the HLA molecules, all these data together indicate that the presence of an infiltrate is a bad prognostic parameter in uveal melanoma. Several studies have shown that UM contain Foxp3-positive regulator cells, which may inhibit effective CTL function [12,55]. An interesting option is to specifically deplete these regulator T cells. A recent study in a mouse model showed that attacking a T-reg specific kinase may help bring back CTLs, which subsequently kill the tumor cells [56]. As T cells may produce factors that bring in macrophages [54], T-cell depletion may also reduce macrophages density, and this may subsequently influence the development of the primary tumor or metastases, as the presence of macrophages is related to angiogenesis and most of the macrophages in UM are of the pro-angiogenic M2 type [40]. This and other T-cell based therapies will only work when there is a proper functioning HLA system present, which we have shown here to be the case.

**Fig 4:**
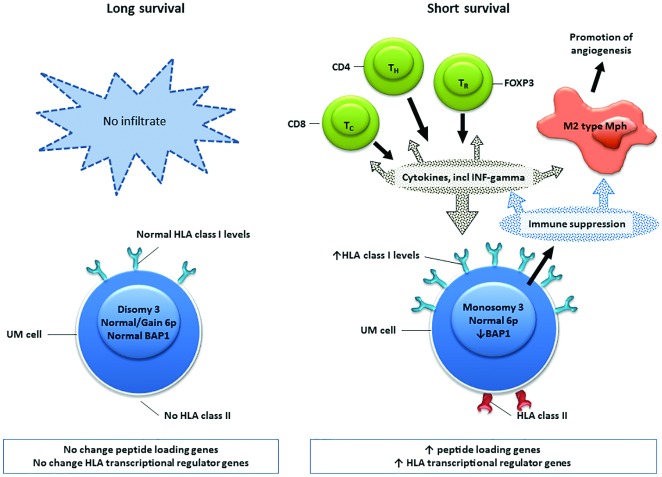
Schematic illustration of tumor characteristics and infiltrate. UM with monosomy 3 attract an infiltrate, producing different cytokines, including Interferon-gamma. The tumor cell (UM cell) responds by increasing HLA class I and II levels, as well as rendering the infiltrating immune cells ineffective (immune suppression) and creating a tumor-favorable environment, with amongst others, stimulation of angiogenesis.

In summary, we show that in primary UM, there is no gene-dose effect of chromosome 6p with regard to HLA expression nor a general abnormal internal regulation of HLA expression. We provide evidence for a co-regulated upregulation of genes encoding for HLA transcriptional regulators, peptide-loading machinery, and HLA proteins on the cell surface, which we attribute to the presence of tumor-infiltrating T cells, secreting IFNG (Fig 4). This indicaties a proper function antigen presenting system, which one would not expect, as likely the most common method a tumor deploys for immune evasion is reducing their antigen expression by down regulation of HLA molecules [57,58] or altering the subtypes of HLA molecules expressed [59]. In earlier work we also showed that this latter is not the case [30]. Together, our data indicates that UM contain a proper functioning HLA antigen expression system, which should allow T-cell mediated tumor cell killing.

## Supporting Information

S1 Fig Chromosome 3 status and gene-expression (Illumina array data) of several HLA regulators. Only significant p-values are shown, all other comparisons between the groups were not significant (p-values not shown). Error-bars represent the interquartile range. Results were obtained using the Mann-Whitney U tests.
(TIF)

S1 Table An overview of the primers used for the validation with qPCR. (DOCX)

S2 Table Percentage of tumor cells staining positive for the monoclonal antibodies for HLA-A, HLA-B or HLA-DR. (DOCX)

S3 Table Hazard ratio’s (Univariate Cox-regression) of HLA gene expression as determined by the Illumina array. (DOCX)

S4 Table Characteristiscs of study population. Baseline characteristics of patients and histological of 28 cases of primary UM, obtained by enucleation, the gene expression values of the indicated genes as determined by the Illumina array, and the p values for associations between the clinical/histopathological characteristics the gene expression values.
(DOCX)

S5 Table qPCR confirmation of the correlations found with the Illumina data, between expression values of HLA regulator genes and TAP1/TAP2, with HLA genes. (DOCX)

S6 Table Changes in mean expression of gene expression levels for markers identifying infiltrating cells, HLA genes, HLA regulator genes, peptide-loading machinery genes, and cytokines. The gene-expression was determined with the Illumina array in 12 primary human uveal melanoma/metastases and their corresponding xenograft in a mouse.
(DOCX)
