# Supplementary figures and images for: Upregulation of HLA Expression in Primary Uveal Melanoma by Infiltrating Leukocytes
Source: PLoS One. 2016 Oct 20;11(10):e0164292. doi: 10.1371/journal.pone.0164292 (PMC5072555; doi:10.1371/journal.pone.0164292)

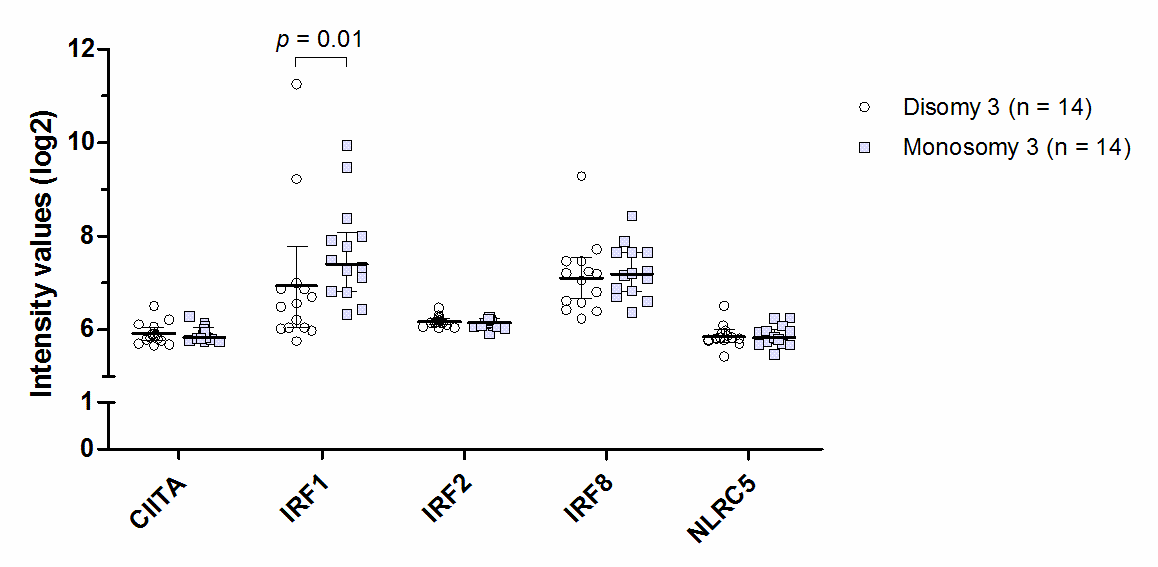

Supplement: S1 Fig — Only significant p-values are shown, all other comparisons between the groups were not significant (p-values not shown). Error-bars represent the interquartile range. Results were obtained using the Mann-Whitney U tests. (TIF) [file pone.0164292.s001.tif]
