# Supplementary material for: Upregulation of HLA Expression in Primary Uveal Melanoma by Infiltrating Leukocytes
Source: PLoS One. 2016 Oct 20;11(10):e0164292. doi: 10.1371/journal.pone.0164292 (PMC5072555; doi:10.1371/journal.pone.0164292)
Supplement: S1 Table — (DOCX) [file pone.0164292.s002.docx]

**S1 Table. An overview of the primers used for the validation with qPCR.**

| Primers | Forward | Reverse |  |
| --- | --- | --- | --- |
| *Beta-Actin* | CGGGACCTGACTGACTACCTC | CTCCTTAATGTCACGCACGATTTC |  |
| *GAPDH* | GCACAGCCCACAGGTTTC | CAGGCGGAGGACAGGATG |  |
| *RPL13* | GTACGCTGTGAAGGCATCAAC | GGTTGGTGTTCATCCGCTTG |  |
| *RPS11* | AAGCAGCCGACCATCTTTCA | CGGGAGCTTCTCCTTGCC |  |
| *HLA-A* | TGTGTTCGTGTAGGCATA | TTGAGACAGAGATGGAGAC |  |
| *HLA-B* | CTCCATCTCTGTCTCAACTT | CATCAACCTCTCATAGCA |  |
| *B2M* | TGCTGTCTCCATGTTTGATGTATCT | TCTCTGCTCCCCACCTCTAAGT |  |
| *HLA-DR* | CAAAGAAGGAGACGGTCTGG | GGCTCTCTCAGTTCCACAGG |  |
| *HLA-DQ* | TGATGGAGATGAGCAGTTC | GCAGCGGTAGAGTTGTAG |  |
| *CIITA(-PIII)* | GCTGGGATTCCTACACAATGC | TCTCCAGCCAGGTCCATCTG |  |
| *IRF1* | TCACCAAGAACCAGAGAA | TCCATCAGAGAAGGTATCAG |  |
| *NLRC5* | CTGGAGGAGTTGATGCTT | GATGGCTGAATGGTAGGT |  |
| *TAP1* | CGAAGCCCAGAAGTTTAG | CCACCAATGTAGAGGATTC |  |
| *TAP2* | CTATTCTGGTCGTGTGATTG | CTGTCTTAGTCTCCTGGAA |  |
