# Supplementary material for: Upregulation of HLA Expression in Primary Uveal Melanoma by Infiltrating Leukocytes
Source: PLoS One. 2016 Oct 20;11(10):e0164292. doi: 10.1371/journal.pone.0164292 (PMC5072555; doi:10.1371/journal.pone.0164292)
Supplement: S2 Table — (DOCX) [file pone.0164292.s003.docx]

**S2 Table. Percentage of tumor cells staining positive for the monoclonal antibodies for HLA-A, HLA-B or HLA-DR**

| Tumor nr. | mAb HCA2 (HLA-A) | mAb HC10 (HLA-B/C) | mAb Tal.1B5 (HLA-DR) |
| --- | --- | --- | --- |
| 01-042 | 0 | 5 | 10 |
| 01-074 | 40 | 25 | 60 |
| 01-091 | 50 | 60 | 10 |
| 01-129 | 80 | 100 | 30 |
| 01-131 | 50 | 60 | 20 |
| 02-158 | 0 | 0 | 5 |
| 02-167 | 70 | 90 | 15 |
| 02-174 | 20 | 70 | 5 |
| 02-199 | 30 | 10 | 15 |
| 03-086 | 30 | 5 | 25 |
| 03-087 | 5 | 0 | 5 |
| 03-120 | 50 | 70 | 100 |
| 03-129 | 60 | 5 | 10 |
| 04-018 | 20 | 10 | 15 |
| 04-035 | 80 | 70 | 15 |
| 04-074 | 5 | 5 | 10 |
| 04-075 | 60 | 50 | 25 |
| 04-103 | 20 | 10 | 5 |
| 04-112 | 50 | 40 | 10 |
| 20-005 | 90 | 60 | 10 |
| 20-042 | 25 | 25 | 5 |
| 20-125 | 10 | 0 | 5 |
| 20-128 | 50 | 40 | 50 |
| 20-173 | 40 | 5 | 80 |
| 20-178 | 25 | 10 | 35 |
| 99-184 | 5 | 10 | 20 |
| 99-187 | 5 | 0 | 10 |
| 99-239 | 95 | 80 | 30 |
