# Supplementary material for: Upregulation of HLA Expression in Primary Uveal Melanoma by Infiltrating Leukocytes
Source: PLoS One. 2016 Oct 20;11(10):e0164292. doi: 10.1371/journal.pone.0164292 (PMC5072555; doi:10.1371/journal.pone.0164292)
Supplement: S3 Table — (DOCX) [file pone.0164292.s004.docx]

**S3 Table. Hazard ratio’s (Univariate Cox-regression) of death due to metastasis for HLA gene expression as determined by the Illumina array.**

| Gene-expression array log2 intensity values (n=28) | Mean (median) | ±SD | HR (*p*-value) | 95% conf. interval |  |
| --- | --- | --- | --- | --- | --- |
| *HLA-A* | 11.3 (11.4) | 0.8 | 2.7 (0.003) | 1.4-5.2 |  |
| *HLA-B* | 10.7 (10.8) | 1.2 | 1.7 (0.004) | 1.2-2.4 |  |
| *B2M* | 12.2 (12.2) | 0.9 | 2.1 (0.01) | 1.2-3.7 |  |
|  |  |  |  |  |  |
| *HLA-DR* | 9.0 (8.9) | 0.9 | 1.4 (0.16) | 0.9-2.4 |  |
| *HLA-DQ* | 7.3 (7.1) | 1.0 | 1.3 (0.21) | 0.9-1.9 |  |

HR = hazard ratio
