# Supplementary material for: Upregulation of HLA Expression in Primary Uveal Melanoma by Infiltrating Leukocytes
Source: PLoS One. 2016 Oct 20;11(10):e0164292. doi: 10.1371/journal.pone.0164292 (PMC5072555; doi:10.1371/journal.pone.0164292)
Supplement: S4 Table — Baseline characteristics of patients and histological of 28 cases of primary UM, obtained by enucleation, the gene expression values of the indicated genes as determined by the Illumina array, and the p values for associations between the clinical/histopathological characteristics the gene expression values. (DOCX) [file pone.0164292.s005.docx]

**S4 Table. Characteristiscs of study population, with hazard ratio’s (HR) for death due to metastasis**

|  | **Baseline Data** | | | |  | **Associations with gene-expr. (mean)** | | | | |
| --- | --- | --- | --- | --- | --- | --- | --- | --- | --- | --- |
| **Categorical variables** | **N** | **%** | **HR (*p*-value)** | **95% conf. interval** |  | ***HLA-A*** | ***HLA-B*** | ***B2M*** | ***HLA-DR*** | ***HLA-DQ*** |
| Patient gender |  |  |  |  |  |  |  |  |  |  |
| Male | 15 | 54 | 0.7 (0.60) | 0.3-2.2 |  | 11.3 | 10.8 | 12.1 | 9.1. | 7.5 |
| Female | 13 | 46 | 1.3 (0.60) | 0.4-4.0 |  | 11.3 | 10.7 | 12.3 | 8.8 | 7.1 |
| *p-values* |  |  |  |  |  | *1.00* | *0.72* | *0.50* | *0.47* | *0.41* |
| Prognostic groups |  |  |  |  |  |  |  |  |  |  |
| Stage I & IIA | 8 | 28 | - |  |  | 11.1 | 10.6 | 11.9 | 8.9 | 7.3 |
| Stage IIB | 10 | 36 | 4.4 (0.18) | 0.5-30.6 |  | 11.3 | 10.6 | 12.2 | 9.0 | 7.3 |
| Stage IIIA | 8 | 29 | 14.1 (0.01) | 1.7-117.9 |  | 11.6 | 11.3 | 12.7 | 9.1 | 7.2 |
| Stage IIIB | 2 | 7 | 5.1 (0.25) | 0.3-82.8 |  | 11.1 | 10.2 | 12.0 | 9.2 | 7.7 |
| *p-values* |  |  |  |  |  | *0.33* | *0.43* | *0.25* | *0.76* | *0.60* |
| Cell type |  |  |  |  |  |  |  |  |  |  |
| Spindle | 10 | 36 | 0.2 (0.44) | 01-1.0 |  | 11.0 | 10.3 | 11.9 | 8.9 | 7.2 |
| Mixed/Epithelioid | 18 | 64 | 4.7 (0.44) | 1.0-21.6 |  | 11.5 | 11.0 | 12.4 | 9.0 | 7.4 |
| *p-values* |  |  |  |  |  | *0.09* | *0.13* | *0.21* | *0.91* | *0.83* |
| Chromosome 3 |  |  |  |  |  |  |  |  |  |  |
| Normal | 14 | 50 | 0.04 (0.003) | 0.01-0.35 |  | 10.1 | 10.1 | 11.8 | 8.9 | 7.2 |
| Loss | 14 | 50 | 22.6 (0.003) | 2.9-176.1 |  | 11.7 | 11.4 | 12.6 | 9.1 | 7.4 |
| *p-values* |  |  |  |  |  | *0.002* | *0.001* | *0.01* | *0.35* | *0.23* |
| Chromosome 6p (HLA loci) |  |  |  |  |  |  |  |  |  |  |
| Normal | 20 | 71 | 6.0 (0.09) | 0.8-46.4 |  | 11.4 | 11.0 | 12.4 | 9.1 | 7.4 |
| Gain | 8 | 29 | 0.2 (0.09) | 0.02-1.3 |  | 11.2 | 10.1 | 11.7 | 8.7 | 7.0 |
| *p-values* |  |  |  |  |  | *0.47* | *0.049* | *0.09* | *0.38* | *0.47* |
|  |  |  |  |  |  | **Associations with gene-expr. (*p-*value)** | | | | |
| **Numerical variables** | **Mean (median)** | **±SD** | **HR (*p*-value)** | **95% conf. interval** |  | ***HLA-A*** | ***HLA-B*** | ***B2M*** | ***HLA-DR*** | ***HLA-DQ*** |
| Age at enucleation (years) (n=28) | 62 (68) | 15 | 1.0 (0.26) | 1.0-1.1 |  | 0.046 | 0.008 | 0.001 | 0.02 | 0.15 |
| LBD (mm) (n=28) | 14 (15) | 3 | 1.4 (0.01) | 1.1-1.7 |  | 0.03 | 0.04 | 0.01 | 0.02 | 0.01 |
| Prominence (mm) (n=28) | 7 (7) | 3 | 1.1 (0.58) | 0.9-1.3 |  | 0.99 | 0.62 | 0.77 | 0.86 | 0.89 |

*Baseline characteristics of patients and histological of 28 cases of primary UM, obtained by enucleation. The gene expression values of the indicated genes were determined by the Illumina array.*

*Tumor size (Largest Basal Diameter and Prominence) has been an important prognostic factor for a long time.*

*LBD = Largest Basal Diameter; HR = hazard ratio (Univariate cox regression).*

*Wilcoxon rank sum test (Mann-Whitney U) was used for comparing two categories, and the Kruskal Wallis Test was used for comparing more than two categories. P-values for associations with numerical data were calculated with Spearman’s correlation (two tailed).*
