# Supplementary material for: Upregulation of HLA Expression in Primary Uveal Melanoma by Infiltrating Leukocytes
Source: PLoS One. 2016 Oct 20;11(10):e0164292. doi: 10.1371/journal.pone.0164292 (PMC5072555; doi:10.1371/journal.pone.0164292)
Supplement: S5 Table — (DOCX) [file pone.0164292.s006.docx]

**S5 Table.** **qPCR confirmation of the correlations found with the Illumina data, between expression values of HLA regulator genes and TAP1/TAP2, with HLA genes.**

|  | *HLA-A* | | *HLA-B* | | *B2M* | | *HLA-DR* | | *HLA-DQ* | | |
| --- | --- | --- | --- | --- | --- | --- | --- | --- | --- | --- | --- |
| HLA transcriptional regulators | *r* | *p* | *r* | *p* | *r* | *p* | *r* | *p* | | *r* | *p* |
| *CIITA* | .437 | 0.02 | .626 | <0.001 | .461 | 0.02 | .615 | <0.001 | | .430 | 0.02 |
| *IRF1* | .527 | 0.001 | .440 | 0.02 | .591 | 0.001 | .819 | <0.001 | | .601 | 0.001 |
| *NLRC5* | .331 | 0.09 | .177 | 0.37 | .363 | 0.06 | .544 | 0.003 | | .539 | 0.003 |
|  |  | |  | |  | |  |  | |  |  |
|  | *HLA-A* | | *HLA-B* | | *B2M* | |  |  | |  |  |
| Peptide loading machinery | *r* | *p* | *r* | *p* | *r* | *p* |  |  | |  |  |
| *TAP1* | .824 | <0.001 | .744 | <0.001 | .878 | <0.001 |  |  | |  |  |
| *TAP2* | .537 | 0.003 | .446 | 0.02 | .536 | 0.003 |  |  | |  |  |

Underlined values confirmed the IIllumina-array results.

*r* = two-tailed Spearman correlation coefficient.

*p* = p-value.
