# Supplementary material for: Upregulation of HLA Expression in Primary Uveal Melanoma by Infiltrating Leukocytes
Source: PLoS One. 2016 Oct 20;11(10):e0164292. doi: 10.1371/journal.pone.0164292 (PMC5072555; doi:10.1371/journal.pone.0164292)
Supplement: S6 Table — The gene-expression was determined with the Illumina array in 12 primary human uveal melanoma/metastases and their corresponding xenograft in a mouse. (DOCX) [file pone.0164292.s007.docx]

**S6 Table.** **Changes in mean expression of gene expression levels for markers identifying infiltrating cells, HLA genes, HLA regulator genes, peptide-loading machinery genes, and cytokines.**

| Marker | *Human tumor* | *Xenograft* | *Delta*  *Human tumor-Xenograft* | *SD*  *Human tumors* | *SD xenografts* | *P-values* |
| --- | --- | --- | --- | --- | --- | --- |
| *CD3D* | 6.39 | 5.05 | 1.35 | 1.12 | 0.22 | < 0.001 |
| *CD3E* | 5.84 | 4.57 | 1.27 | 1.07 | 0.15 | < 0.001 |
| *CD4* | 7.14 | 5.90 | 1.24 | 0.75 | 1.09 | < 0.001 |
| *CD8A* | 7.52 | 6.61 | 0.91 | 0.84 | 0.16 | 0.001 |
| *CD8B* | 5.30 | 5.17 | 0.13 | 0.26 | 0.11 | 0.34 |
| *CD68* | 10.62 | 9.22 | 1.39 | 0.60 | 1.05 | < 0.001 |
| *CD163* | 6.08 | 4.60 | 1.48 | 0.75 | 0.14 | < 0.001 |
| *HLA-A* | 11.54 | 10.54 | 1.0 | 0.59 | 1.15 | 0.005 |
| *HLA-B* | 12.44 | 10.45 | 1.99 | 0.77 | 1.76 | < 0.001 |
| *B2M* | 10.98 | 9.68 | 1.30 | 0.72 | 0.92 | 0.004 |
| *HLA-DRA* | 10.44 | 4.94 | 5.50 | 1.45 | 0.15 | < 0.001 |
| *HLA-DQA1* | 7.25 | 4.18 | 3.07 | 2.09 | 0.19 | < 0.001 |
| *HLA-DQA2* | 5.41 | 4.82 | 0.59 | 0.41 | 0.23 | 0.002 |
| *IRF1* | 7.61 | 6.38 | 1.23 | 0.87 | 0.23 | < 0.001 |
| *IRF2* | 9.21 | 8.85 | 0.36 | 0.37 | 0.38 | 0.046 |
| *IRF8* | 7.09 | 5.43 | 1.66 | 0.84 | 0.17 | < 0.001 |
| *CIITA* | 7.46 | 6.24 | 1.22 | 0.77 | 0.22 | < 0.001 |
| *NLRC5* | 7.80 | 6.89 | 0.90 | 0.67 | 0.36 | 0.002 |
| *PDIA3* | 7.02 | 7.03 | -0.01 | 0.25 | 0.22 | 0.93 |
| *TAP1* | 10.13 | 8.76 | 1.36 | 1.04 | 0.99 | 0.005 |
| *TAP2* | 8.88 | 8.13 | 0.75 | 0.74 | 0.52 | 0.02 |
| *Tapasin* | 10.30 | 9.58 | 0.72 | 0.49 | 0.56 | 0.004 |
| *Calreticulin* | 10.78 | 10.83 | -0.05 | 0.16 | 0.17 | 0.54 |
| *IFNG* | 4.11 | 3.85 | 0.26 | 0.29 | 0.11 | 0.005 |
| *CCL2* | 7.87 | 5.93 | 1.94 | 1.33 | 0.81 | < 0.001 |
| *TGFB1* | 7.84 | 6.91 | 0.92 | 0.54 | 0.63 | < 0.001 |
| *TGFB2* | 6.96 | 6.77 | 0.19 | 0.78 | 1.41 | 0.14 |
| *TGFB3* | 7.04 | 6.55 | 0.49 | 0.77 | 0.41 | 0.08 |
| *TNF* | 5.79 | 5.71 | 0.08 | 0.35 | 0.17 | 0.40 |

*The gene-expression was determined with the Affymetrix array in 12 primary human uveal melanoma/metastases and their corresponding xenograft in a mouse.*

*P-values have been calculated with the Wilcoxon-signed rank test.*
